# Cross-feeding and co-degradation within a bacterial consortium dominated by challenging-to-culture *Leucobacter* sp. HA-1 enhances sulfonamide degradation

**DOI:** 10.1128/aem.00590-25

**Published:** 2025-06-24

**Authors:** Guoqiang Zhao, Houyu Yu, Juanjuan Wang, Bo Jiang, Fangya Zhong, Rui Zhang, Tianzhi Jiang, Mo Yang, Hui Wang, Xing Huang

**Affiliations:** 1 Department of Microbiology, College of Life Sciences, Nanjing Agricultural University 70578, Nanjing, Jiangsu, China; 2 Jingzhou Municipal Ecological Environment Information and Assessment Center, Jingzhou, Hubei, China; 3 State Key Laboratory of Biocatalysis and Enzyme Engineering, School of Life Sciences, Hubei University 12563, Wuhan, Hubei, China; Shanghai Jiao Tong University, Shanghai, China

**Keywords:** sulfonamides, cross-feeding, co-degradation, *Leucobacter *sp. HA-1, interaction mechanism

## Abstract

The use of sulfonamides (SAs) leads to residual environmental pollution. Bacteria play a crucial role in the degradation of SAs, and microbial consortium offers advantages over single bacterium. However, the complexity of the degradation processes and interaction mechanisms within such consortia remains a mystery. Here, a consortium named ACJ, comprising *Leucobacter* sp. HA-1, *Bacillus* sp. HC-1, and *Gordonia* sp. HAEJ-1, isolated from activated sludge in the wastewater treatment facilities of pharmaceutical plants, was identified as capable of degrading various SAs. Here, a pure culture of *Leucobacter* sp. HA-1, which plays a key role in SAs degradation, was obtained with the auxotrophic requirements (ARs) provided by strains HC-1 and HAEJ-1. Strain HA-1 initiated the breakdown of SAs molecules, releasing heterocyclic structure products and trihydroxybenzene (HHQ), which were further degraded and used for growth by strain HAEJ-1. Genomic, transcriptomic, and metabolomic analyses indicated that genes related to nucleotide repair, ABC transporters, quorum sensing, the TCA cycle, and the cell cycle in strain HA-1 were upregulated during co-culture compared to cultures without the other two strains, which indicated that certain factors of strains HC-1 and HAEJ-1 activated the growth of strain HA-1. These results demonstrate a bidirectional ecological relationship of cross-feeding and co-degradation among the consortium members. Overall, this study provides new insights into the mechanisms of microbial interaction and co-degradation in sulfonamides-contaminated environments.

**IMPORTANCE:**

Sulfonamides (SAs) are widely used antibiotics that have significantly harmed the ecological environment, emerging as a new environmental pollutant. Currently, limited research exists on the mechanisms of microbial consortium interaction and co-degradation of environmental pollutants. Addressing challenges in environmental pollutant degradation, this study isolated a bacterial consortium, ACJ, dominated by the challenging-to-culture *Leucobacter* sp. HA-1 from a sewage treatment plant and unveiled their interaction and co-degradation mechanisms during SAs degradation. Toxicological experiments demonstrated that the degradation of SAs by consortium ACJ substantially reduced environmental damage. These findings offer new insights into the collaborative mechanisms of the consortium of environmental pollutant-degrading microbial consortia and provide valuable microbial resources for the remediation of antibiotic-contaminated environments.

## INTRODUCTION

Sulfonamides (SAs) constitute a significant class of antibiotic medications, with sulfaquinoxaline (SQX) being an important example, widely used to treat infections in both humans and livestock (1, 2). However, their inherent stability and persistence have resulted in their widespread detection in various aquatic and soil environments, thereby posing substantial ecological health risks (3–5). The extended presence of SAs in the environment has also been linked to the dissemination of antibiotic resistance genes (ARGs) and toxicity to non-target organisms (6–8). Consequently, the effective removal of SAs residues in the environment is imperative to mitigate ecological health hazards.

Microbial degradation emerges as a promising, nature-based, sustainable, and environmentally friendly removal of SAs residues approach (9, 10). Previous investigations have identified various SAs-degrading bacterial consortia, including those consisting of *Achromobacter denitrificans* PR1 and *Leucobacter* sp. GP (11), *Nocardioides*, *Acidovorax*, and *Sphingobium* (12), *Paenarthrobacter ureafaciens* YL1 and *Pseudomonas koreensis* YL2 (13), *Paenarthrobacter* sp. P27 and *Norcardioides* sp. N27 (14), *Paracoccus denitrificans* and *Shewanella oneidensis* MR-1 (15). Each of these SAs degradable bacterial consortia contains different strains and may lead to diverse pollutant metabolic pathways (16–18). The efficiency and pathways of degradation varied among different strains or consortia when addressing various types of SAs (17, 19). The residues and degradation products from the biodegradation process may still pose toxic effects on the environment and organisms, affecting the metabolic activity and survival ability of organisms in the environment (20, 21). However, previous studies have primarily focused on the removal mechanisms and transformation pathways during SAs biodegradation, while overlooking the potential toxicity and environmental risks of the degradation products (22, 23). Although bacterial consortia involved in SA degradation have been isolated and characterized, the specific degradation and metabolic roles of individual strains within these consortia remain to be clarified.

Microbial communities exhibit bidirectional or unidirectional interactions to establish various relationships, including synergy, symbiosis, reciprocity, and competition (24–26). These relationships are sustained primarily through the sharing of metabolites resulting from biosynthesis and metabolism among community members (24, 27). For example, intermediate products generated from bacterial degradation of pollutants can be metabolized and utilized by the other community members, supporting the physiological functions of the entire bacterial community (28, 29). Research indicates the presence of cross-feeding relationships in microbial communities, particularly in metabolic pathways such as amino acid, peptide, and vitamin synthesis and metabolism, carbohydrate synthesis and metabolism, organic acid metabolism, and pollutant degradation. These pathways enhance the growth and metabolic efficiency of individual members (30–33). However, microbial communities in different environments can establish a variety of complex ecological relationships that require further exploration. *Leucobacter* sp., frequently found in the SAs degrading consortium, harbors the flavin-dependent monooxygenase *sadA*, responsible for the initial cleavage of SAs. This implies a significant role for *Leucobacter* sp. in SAs degradation (34). However, the inability to obtain a pure culture of this bacterium in the consortium has significantly constrained investigations into its role in microflora (11, 35). Further elucidation is needed to reveal the division of labor and specific interactions within consortium members during SA degradation and co-culture processes.

Here, we isolated a microbial consortium named ACJ from activated sludge in wastewater treatment facilities of pharmaceutical plants, capable of degrading various SAs. This study aims to (i) investigate the degradation efficiency of ACJ toward various SAs, analyze the metabolic pathway, and evaluate the toxicity of degradation products; (ii) obtain a pure culture of *Leucobacter* sp. HA-1 and analyze the co-degradation relationship of SQX by the consortium ACJ; (iii) analyze the gene responses and cross-feeding mechanisms of each member in the ACJ co-culture through the transcriptome and metabolome analysis.

## MATERIALS AND METHODS

### Chemicals and medium

Sulfaquinoxaline (SQX), sulfamethazine (SMZ), sulfamethoxazole (SMX), sulfathiazole (ST), sulfapyridine (SP), sulfafurazole (SIZ), 2-aminoquinoxaline (2-AQ), 2-amino-4,6-dimethylpyrimidine (DMPA), 3-amino-5-methylisoxazole (3A5MI), trihydroxybenzene (HHQ) were purchased from Beijing J&K Scientific Co., Ltd; these chemicals were dissolved in methanol, an organic reagent, and configured to the appropriate concentration for use. All strains were cultured in the Lysogeny Broth medium (LB, 1 L) consisting of 5.0 g yeast extract, 10.0 g tryptone, and 10.0 g NaCl, pH 7.0. The strains were cultured in 1/10 LB medium for transcriptome and metabolome sequencing experiments. Unless otherwise stated, the most degradation experiments were carried out in Mineral Salt Medium (MSM, 1 L) containing NH_4_Cl 1.0 g, KH_2_PO_4_ 0.5 g, K_2_HPO_4_ 1.5 g, NaCl 1.0 g, and MgSO_4_⋅7H_2_O 0.2 g, without the trace element solution. To verify the degradation function of strain HA-1, the degradation experiment of strain HA-1 was conducted in a mixed medium of LB and MSM.

### Strain isolation and SA degradation

Activated sludge was collected from the wastewater treatment facilities of pharmaceutical plants in Jiangling County, Jingzhou, Hubei Province, China (112°26'30"E, 30°2'13"N), and supplemented with an appropriate concentration of SQX for continuous enrichment in MSM medium. The degradation of SQX was monitored using a UV spectrophotometer. The enriched culture showing significant degradation was diluted and plated on an LB solid plate containing 200 mg/L of SQX and incubated at 30°C. Single colonies were selected, and 16S rRNA gene PCR was conducted on each strain using 27F and 1492R (36). The PCR and plasmid construction followed protocols from relevant literature with some modifications (37). The 16S rRNA sequencing results were used to construct the strain’s phylogenetic tree through online sequence alignment (38). The strains isolated from the collection plate were subjected to a process of washing with sterile water, after which they were introduced into LB medium for further enrichment, the strains were cultured at a temperature of 30°C for 2 days. The bacteria were harvested by centrifugation, washed in MSM medium twice, and resuspended to assess the degradation properties of the strains toward SAs. The degradation characteristics of various SAs, as well as the effects of temperature and pH, were examined by incorporating a concentration of 50 mg/L of SAs into 20 mL of MSM medium, followed by the addition of bacterial suspensions with an optical density at 600 nm (OD_600_) of 0.5, and incubating the samples at 30°C. Samples were collected at intervals of 0, 3, and 5 days, and the degradation of SAs was analyzed using high-performance liquid chromatography (HPLC). Three SAs (SQX, SMZ, and SMX), known for their high pollution levels of pollution, were selected for this study using a consistent methodology. The experimental conditions varied with temperatures set at 20, 30, 35, and 40°C, and pH levels adjusted to 5.0, 6.0, 7.0, 8.0, and 9.0. After 5 days, samples were collected to determine the degradation rates of the SAs. To explore the correlation between these rates and bacterial growth, a bacterial suspension with an OD_600_ of 0.5 was inoculated, degrading SQX, SMZ, and SMX at a final concentration of 50 mg/L at 30°C and pH 7.0. Samples were collected in a bi-daily manner over a 7-day period to assess both SA degradation and bacterial growth. Each trial was replicated thrice.

For quantitative analysis of SA degradation, 500 µL of the reaction solution was extracted during the experiment, and an equal volume of methanol was added to halt the reaction. Following high-speed agitation for 2 min, the upper liquid phase was centrifuged at 12,000 × *g* for 1 min. The supernatant was then filtered through a 0.22 µm membrane into a brown vial for analysis under uniform HPLC conditions. The analysis employed a Thermo Scientific Ultimate 3000 HPLC system equipped with a Thermo Scientific Acclaim 120 C18 reverse column (5 µm, 120 A, 4.6 × 250 mm). The mobile phase was composed of acetonitrile, water, and acetic acid, with a volumetric ratio of 40:59:1 (vol/vol/vol). The column temperature was maintained at 40°C, with a flow rate of 1 mL/min, and detection was conducted at 270 nm with a sample injection volume of 20 µL. Quantification of SAs and their intermediates was performed by constructing a standard curve using the external standard method, correlating the peak area of the sample to the curve.

### Co-degradation experiment

Strains HC-1 and HAEJ-1 were cultivated in 100 mL of LB medium until the logarithmic growth phase was reached. Following this, the extracellular supernatant and bacterial cells were separated by centrifugation at 8,000 × *g* for 10 min and stored at 4°C. The cells were washed twice with MSM medium and resuspended in 20 mL of the same medium. Cells were then disrupted using an ultrasonic breaker for 30 min and centrifuged at 12,000 × *g* at 4°C for 1 h to obtain the intracellular supernatant. Both the extracellular and intracellular supernatants were subjected to filtration thrice through a sterile filter head, following which they were stored at 4°C for subsequent use. To ascertain the optimal conditions for the cultivation of HA-1 in a solo culture, 1 mL of each of the extracellular supernatant and intracellular supernatant from strains HC-1 and HAEJ-1 was introduced into 10 mL of LB medium, and single colonies of HA-1 were inoculated. The growth of HA-1 was monitored at 30°C, with a control of HA-1 alone inoculated in LB medium. Following the same culturing procedure, varying volumes (1, 2, 3, 4, and 5 mL) of intracellular supernatant were introduced to the LB medium, with the MSM medium being adjusted to ensure equivalent total volumes across all cultures. Following this, single colonies of HA-1 were inoculated. The growth of HA-1 was observed from 48 h onward, with SQX administered at 144 h. Cultures were maintained at 30°C for 5 days to assess SQX degradation, and the post-degradation reaction status was documented. To isolate the strain HA-1 alone, 100 µL of the intracellular supernatant was evenly spread on the surface of an LB agar plate. The inoculated HA-1 medium was then spread on the surface of an LB plate coated with intracellular supernatant and incubated at 30°C for 3 days. To verify the functions played by each strain in the consortium ACJ during the degradation process, the strain HA-1 was combined with strains HAEJ-1 and HC-1 to degrade SQX in MSM medium, respectively. A large volume of sterile intracellular supernatant was added to 100 mL of LB medium, and the inoculated strain HA-1 was cultured at 30°C for 5 days, centrifuged at 8,000 × *g* for 10 min, and the HA-1 bacteria were collected, washed twice with MSM medium, and resuspended to prepare the HA-1 bacterial suspension. The MSM medium, containing SQX at a final concentration of 50 mg/L, was supplemented with 1 mL of the HA-1 suspension, maintaining an initial inoculation volume ratio of HA-1 to HC-1 or HAEJ-1 at 1:1 by adding an equal volume of suspension from HC-1 or HAEJ-1 into MSM medium, with an OD_600_ of 0.5 for each strain. The degradation experiment was conducted at a temperature of 30°C, with a control group that did not receive any bacterial suspensions, and samples were collected daily to monitor SQX degradation. The effect of different inoculation volume ratios of HA-1 to HAEJ-1 or HC-1 was tested, including ratios of 1:1, 1:2, 1:3, 2:1, and 3:1, where the proportion of 1 represented an OD_600_ of 0.5. The degradation of SQX was assessed using the same methodology, with samples collected at 3 and 5 days and analyzed using HPLC. Each degradation experiment was replicated in three separate trials. Furthermore, to investigate the contribution of strains HC-1 and HAEJ-1 to the degradation of SQX, individual degradation experiments using these strains were conducted with MSM medium containing 50 mg/L of SQX, 2-AQ, or HHQ, and the degradation process was monitored at 30°C, with samples taken at regular intervals for analysis by HPLC. High-performance liquid chromatography-tandem mass spectrometry (HPLC-MS/MS) was performed on an AB SCIEX Triple TOF 5600 plus high-resolution mass spectrometry using both positive and negative ion modes to identify intermediate metabolites.

In order to verify the growth of strains HC-1 and HAEJ-1 in the co-culture system, the prepared HA-1 bacterial suspension and HC-1 or HAEJ-1 bacterial suspension were mixed and cultured in 1/10 LB medium or 1/10 LB medium with 50 mg/L SQX, respectively, according to the ratio of 1:1. The single culture of strains HC-1 and HAEJ-1 was used as the control, and the final OD_600_ value of each strain in the medium was set at 0.5. The samples were incubated at 30°C, followed by dilution on LB solid plates at 12 h intervals. The biomass of strains HC-1 and HAEJ-1 in the co-culture system was calculated by plate counting. All experiments were conducted in triplicate.

### Genome sequencing and analysis

The bacteria strains HA-1, HC-1, and HAEJ-1 were individually cultured, and their total DNA was extracted using the FastPure Bacteria DNA Isolation Mini Kit from Vazyme Biotech Co., Ltd, Nanjing, following the provided instructions. The bacterial genomes meeting the DNA concentration criteria were sequenced. The genomes of HA-1, HC-1, and HAEJ-1 were sequenced by Shanghai Majorbio Bio-Pharm Technology Co., Ltd. HA-1′s genome was sequenced using Illumina + PacBio (second generation + third generation) technologies, with a minimum of 100 × PacBio sequencing data and 100 × Illumina sequencing data to achieve a comprehensive and accurate assembly, including plasmids. For HC-1 and HAEJ-1, Illumina’s second-generation sequencing platform was utilized to generate 400 bp paired-end reads from qualified DNA samples, with PE150 (paired-end) sequencing involving 150 bp read length for single-end sequencing. Each sample provided a minimum raw data volume of 100 × genome coverage depth, leading to the assembly of multiple genomic scaffolds. The genome analysis involved quality control of original data, statistical analysis, genome evaluation, comparison, assembly, and other steps to obtain specific information on genome structure, functional gene annotation, metabolic pathways, pathogenic systems, and other characteristics of each strain. This analysis aimed to identify potential genes involved in SQX degradation and to facilitate subsequent transcriptome and metabolome sequencing and analysis.

### Transcriptome sequencing and analysis

Six different processing analyses were conducted on the transcriptome data, including HA-1, HC-1, HAEJ-1, HA-1 + HC-1 (group AC), HA-1 + HAEJ-1 (group AJ), and HA-1 + HC-1 + HAEJ-1 (group ACJ). Bacterial suspensions of strains HA-1, HC-1, and HAEJ-1 were cultured and prepared by the same method as above. The same amount of bacterial suspension was added to all treatments so that the final OD_600_ value of each bacterium was 0.5 to prepare the co-culture strain system. The bacterial culture in each treatment was carried out in 1/10 LB medium, and the single bacterial suspension initially inoculated was used as the control. Following a 5-day incubation period at 30℃, the bacteria were centrifuged and harvested, with each sample type being replicated three times. Total RNA extraction was performed using TRIzol reagent as per the manufacturer’s protocol (Invitrogen, Carlsbad, CA), and RNA quantification was carried out using Nanodrop2500 (NanoDrop Technologies, Wilmington, DE, USA). Subsequently, RNASeq transcriptome libraries were prepared using the Illumina TruSeq RNA Sample Preparation Kit (San Diego, CA) and sequenced on the Illumina NovaSeq 6000 platform by Majorbio Biopharm Technology Co., Ltd (Shanghai, China). The raw RNA-Seq data underwent processing using FastQC software and internal Perl scripts to eliminate adapters and low-quality sequences, resulting in clean RNA-Seq data. Clean reads from each sample were aligned to a specified reference genome, and the read count data were normalized. Differentially expressed genes were identified using DESeq2 (1.30.1) with statistical significance set at *P*-adjust <0.05 and |log2 fold change| ≥ 1. Subsequently, the sequences were annotated through gene Ontology (GO) enrichment analysis and Kyoto Encyclopedia of Genes and Genomes (KEGG) pathway enrichment analysis to facilitate functional and metabolic pathway investigations.

### Metabolome analysis

Four different treatments were utilized for comparative metabolomics analysis, namely, HC-1 intracellular supernatant, HAEJ-1 intracellular supernatant, HC-1 intracellular supernatant + HA-1 (group AC), and HAEJ-1 intracellular supernatant + HA-1 (group AJ). These treatments were conducted in 1/10 LB medium to examine the substances from the HC-1 or HAEJ-1 intracellular supernatant utilized by HA-1, and the components of 1/10 LB medium were measured as a control. Each experiment group received the same volume of HA-1 suspensions so that the final OD_600_ value was 0.5, with four replicates performed for each treatment. Following 5-day incubation at 30℃, the supernatant was harvested through centrifugation at 12,000 × *g* for 10 min and transferred to a new centrifuge tube in an equivalent volume. Subsequently, the supernatant was freeze-dried into a powder using a Thermo Heto PowerDry LL3000 machine and sent to Shanghai Majorbio Bio-Pharm Technology Co., Ltd. for metabolomics sequencing. LC-MS/MS analysis was carried out using the Thermo UHPLC-Q Exactive HF-X system with an ACQUITY HSS T3 column (100 mm × 2.1 mm, 1.8 µm, Waters, USA). Mass spectrometry data were acquired on the Thermo UHPLC-Q and the Exactive HF-X mass spectrometer with electrospray ionization (ESI) source operating in positive and negative modes. The raw data were processed and visualized using Progenesis QI software (Waters Corporation, Milford, USA). Metabolites were identified and classified using KEGG for metabolic pathway analysis. Significant differences in metabolites between groups were determined at *P* < 0.05 and VIP > 1.

### Toxicity comparison of SQX degraded by consortium ACJ

Many studies have neglected to evaluate the toxicity of SA antibiotic degradation products. Therefore, the toxicity of these products should be assessed to ensure their safe utilization by microorganisms (21, 39). Final concentrations of SQX were set at 10, 25, 50, 100, and 200 mg/L, and a bacterial suspension was prepared through the co-culture of bacterial consortium ACJ. Degradation experiments were conducted in 20 mL of MSM medium supplemented with this consortium and incubated at 30°C until SQX was completely degraded. A control group included only the bacterial suspension without consortium addition. The supernatant obtained through centrifugation was stored to evaluate whether consortium ACJ could reduce SQX’s toxic effects on organisms. For strain toxicity assessment, *E. coli* DH5α and *Bacillus subtilis* SCK6 were cultured overnight in LB medium. A quantity of 10 µL of the bacterial liquid under consideration was transferred to fresh LB test tubes. These tubes contained different concentrations of SQX and its degradation product, with the total volume of the transfer being 4 mL. After incubating for 10 h at 37°C, the samples were subjected to dilution at various concentrations and subsequently plated onto LB agar plates. The OD_600_ for each strain under each treatment was measured, and the growth inhibition rate was calculated. The experiment was replicated thrice for each strain toxicity test. The seeds of maize and radish were disinfected and soaked in a 10% H_2_O_2_ solution. Thereafter, seeds exhibiting similar characteristics were selected for a 12 h soak in the medium containing SQX and its degradation product. This soaking process was conducted in the absence of light. Seeds soaked in water and methanol served as controls. Twenty seeds were placed in a petri dish with the supernatant and incubated at a constant 25°C in a dark environment to facilitate germination. After 3 days, seed germination was observed, sprout height was measured, and the germination inhibition rate was calculated.

### Data analysis

The peak area of SAs and their degradation products was manually integrated using HPLC, and the degradation efficiency was calculated. Data were processed and analyzed using Microsoft Excel, and graphs were produced with GraphPad Prism version 8.0.2.263 (San Diego, CA, USA). Significance tests for partial results were conducted using one-way ANOVA (*P* < 0.05).

## RESULTS

### Isolation of degrading strains and SA degradation

**Fig 1:**
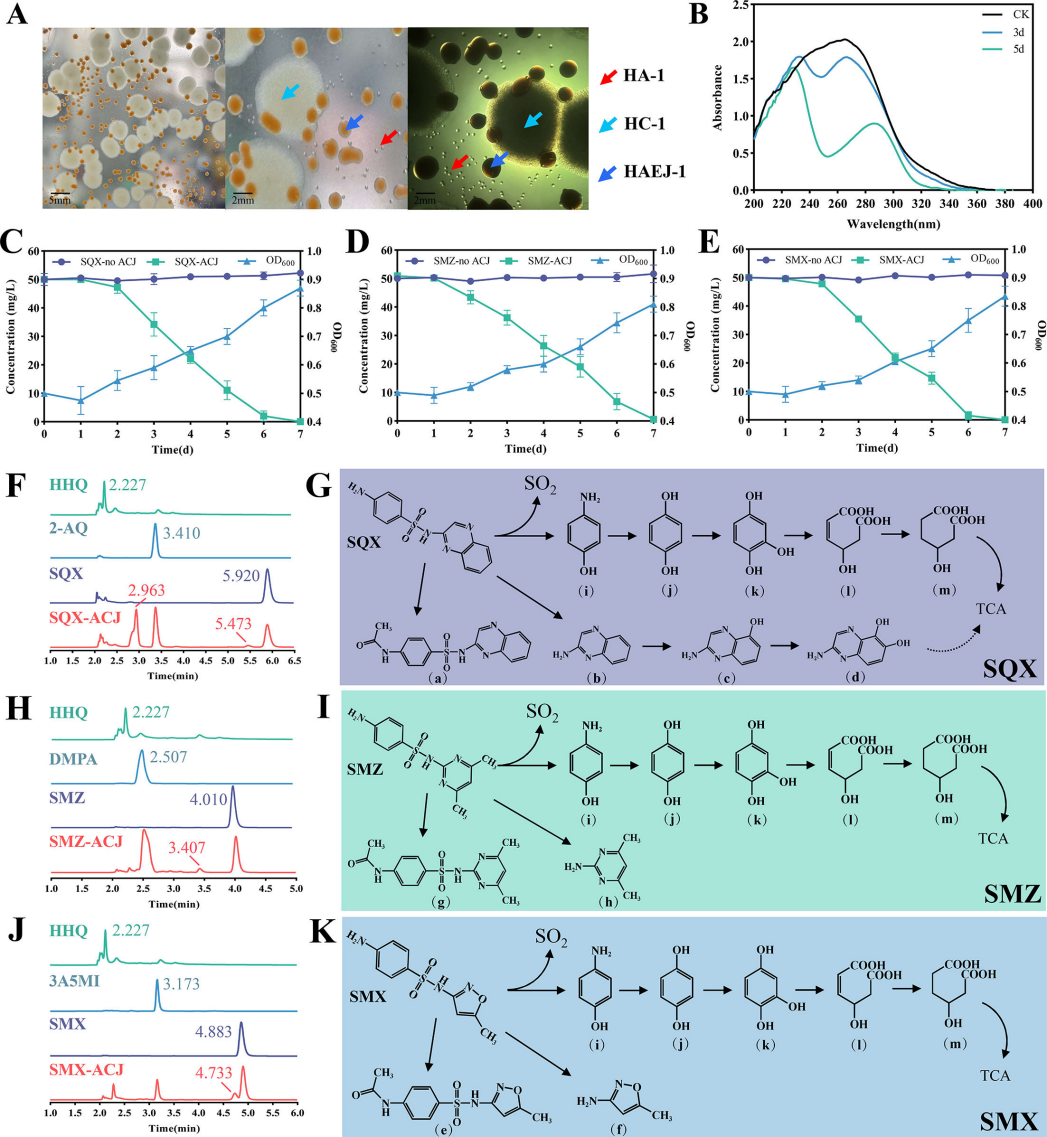
Isolation of strains and degradation of SAs. (A) State of degradation community ACJ composed of HA-1, HC-1, and HAEJ-1 on LB solid plate. (B) Ultraviolet scanning to verify the degradation effect of SAs during the isolation of degrading bacteria. Degradation and growth of SQX (**C**), SMZ (**D**), and SMX (**E**) by ACJ. Degradation of SQX (**F**), SMZ (**H**), and SMX (**J**) was determined by HPLC, and the numbers on the peak chart indicate the peak time of the substance in HPLC. Metabolic pathway of SQX (**G**), SMZ (**I**), and SMX (**K**) degradation by ACJ. a–m represent the metabolic substrate names in HPLC-MS/MS results. There were three replicates in each experiment.

In the initial stage, the degradation capabilities of individual bacteria from an SQX-enriched culture were repeatedly tested on plates. It was observed that the degradation efficiency and HPLC detection results for individual bacteria differed significantly from those of the enriched culture. It was only when all bacterial colonies on the plate were eluted that the degradation of SQX mirrored the phenomenon observed in the enriched culture, suggesting the presence of potential degrading strains within the plate. Microscopic examination identified three distinct bacterial species within the consortium, designated ACJ, which included minute colonies of one bacterium that were difficult to observe (Fig. 1A). Early assessments confirmed ACJ’s ability to degrade SQX, evidenced by noticeable changes in the UV detection peak shape after 3 and 5 days of co-culture degradation (Fig. 1B). Further analyses involved the selection of individual bacterial colonies from ACJ for 16S rRNA gene amplification, combined with morphological observation and phylogenetic analysis, identifying the strains as *Leucobacter* sp. HA-1, *Bacillus* sp. HC-1, and *Gordonia* sp. HAEJ-1, respectively (Fig. S1). The ACJ consortium demonstrated the capacity to degrade various heterocyclic SAs (Fig. S2A). Three SAs (SQX, SMZ, and SMX), known for their high pollution levels and rapid degradation rates, were selected for further investigation of ACJ’s degradation characteristics (1, 2, 12), with optimal conditions determined to be 30℃ and a pH of 7.0 (Fig. S2B and S2C). The bacterial consortium ACJ, comprising three strains, showed rapid degradation and utilization of SAs (Fig. 1C through 1E).

The degradation products of SQX, SMZ, and SMX were identified through the utilization of HPLC-MS/MS methodologies (Fig. 1F, H and J, and S3). Five distinct products were detected in the degradation of SQX, with mass-charge ratios as follows: SQX (RT 1.212 min, *m*/*z* 299.0594), metabolite a (RT 2.101 min, *m*/*z* 341.0691), metabolite b (2-AQ, RT 3.910 min, *m*/*z* 144.0561), metabolite c (RT 3.253 min, *m*/*z* 160.0506), and metabolite d (RT 1,728 min, *m*/*z* 176.0456). Metabolites c and d were identified as further degradation products of 2-AQ, representing a new degradation pathway for SQX. In the cases of SMX and SMZ, three unique substances were detected as degradation products for each: SMX (RT 4.771 min, *m*/*z* 254.0594), metabolite e (RT 6.152 min, *m*/*z* 296.0702), and metabolite f (3A5MI, RT 1.068 min, *m*/*z* 99.0556) for SMX; and SMZ (RT 4.224 min, *m*/*z* 279.0915), metabolite g (RT 5.224 min, *m*/*z* 321.1020), and metabolite h (DMPA, RT 1.564 min, *m*/*z* 124.0873) for SMZ. Additionally, the same degradation products were identified across all three SAs, named metabolite i (RT 1.654 min, *m*/*z* 110.0602), metabolite j (RT 1.146 min, *m*/*z* 111.0440), metabolite k (HHQ, RT 0.784 min, *m*/*z* 125.0353), metabolite l (RT 0.587 min, *m*/*z* 159.0291), and metabolite m (RT 3.402 min, *m*/*z* 161.0528). The metabolic pathways of ACJ for the three types of SAs were thoroughly investigated using standard substances and HPLC-MS/MS analyses (Fig. 1G, I and K).

### Mechanism of consortium ACJ co-degrading SQX

**Fig 2:**
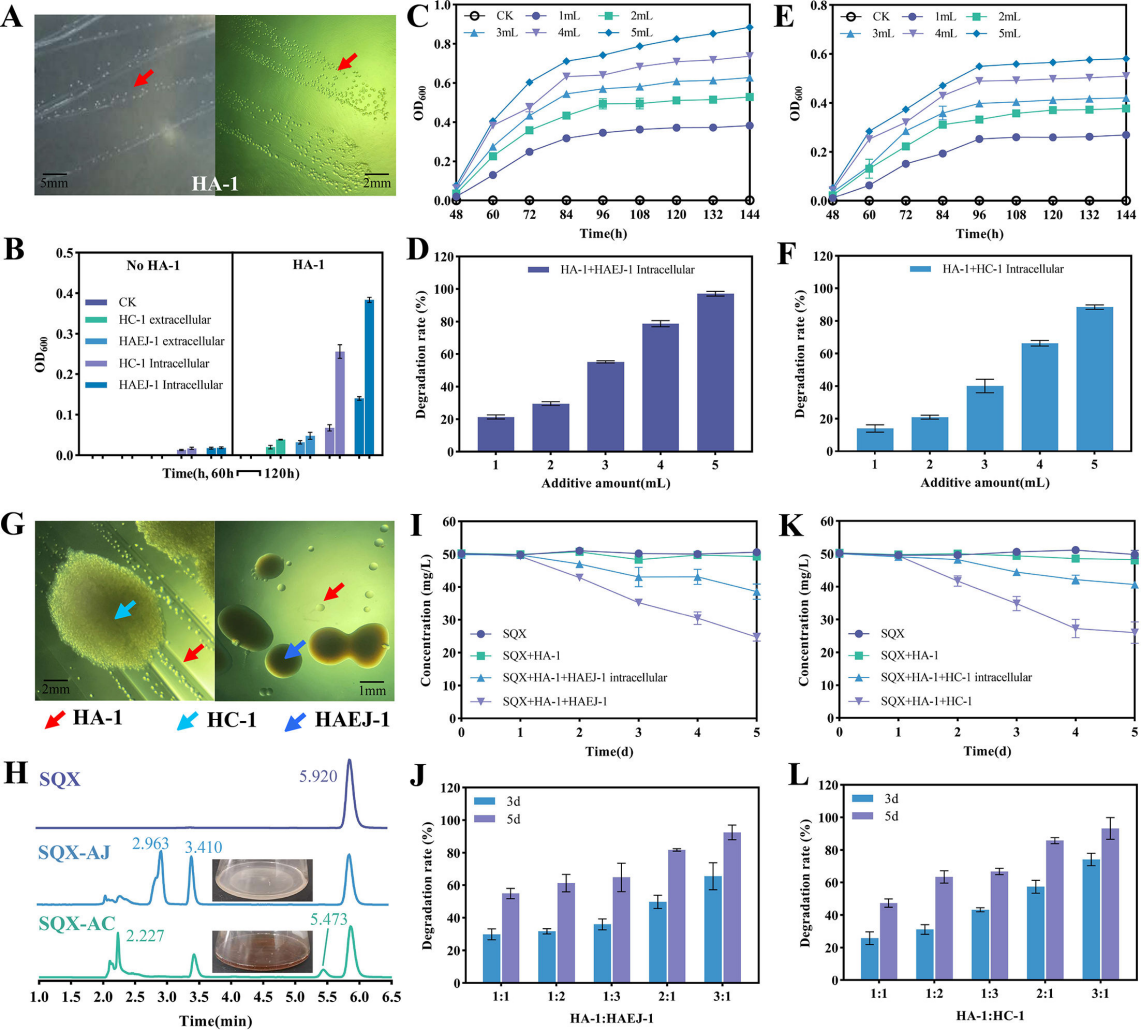
Growth characteristics of challenging-to-cultivate strain HA-1 and consortium ACJ co-degrade SQX. (A) Growth state of strain HA-1 on LB solid plate. HA-1 utilizes the growth of intracellular or extracellular substances of HC-1 or HAEJ-1. (B) HA-1 utilizes the growth of intracellular or extracellular substances of HC-1 or HAEJ-1. Effects of intracellular additions of HAEJ-1 (**C**) and HC-1 (**D**) on the growth of strain HA-1. Effects of intracellular additions of HAEJ-1 (**E**) and HC-1 (**F**) on the degradation of SQX by strain HA-1. (G) State of strain HA-1 on solid LB plates after compounding with HC-1 or HAEJ-1, respectively. (H) HPLC diagram and medium state of degradation of SQX by the combination of AJ (HA-1 and HAEJ-1) and AC (HA-1 and HC-1). Role of HAEJ-1 (**I**) or HC-1 (**K**) in the degradation of SQX by strain HA-1. Effect of the compounding ratio of HA-1 to HAEJ-1 (**J**) or HC-1 (**L**) on the degradation of SQX. There were three replicates in each experiment.

Cultivation experiments on each strain in ACJ revealed that *Leucobacter* sp. HA-1 was nutrient-deficient, which impeded its growth in various media. The present study demonstrated that both the extracellular and intracellular supernatant derived from HC-1 and HAEJ-1, when incorporated into LB medium, were found to be utilized by HA-1 for growth. Furthermore, the investigation established that the intracellular supernatant exerted a more pronounced promoting effect on HA-1 growth than the extracellular supernatant (Fig. 2B). Moreover, HA-1 could be independently cultured on LB solid plates containing the intracellular supernatant from HC-1 or HAEJ-1 (Fig. 2A). It was observed that HA-1 biomass positively correlated with the addition of intracellular components from HC-1 or HAEJ-1, and HA-1 growth stabilized after a period of cultivation (Fig. 2C and D). The degradation rate of SQX by HA-1 increased with the quantity of intracellular supernatant added, and a color change in the medium from light yellow to dark brown was noted (Fig. 2E and F). This relationship between the degradation rate of SQX and the color change of the medium from light yellow to dark brown was observed (Fig. S4).

The interaction between HA-1 co-cultured with HC-1 (group AC) and HA-1 with HAEJ-1 (group AJ) on an LB plate is illustrated in Fig. 2G. HPLC analysis results of SQX degradation by groups AJ and AC are presented in Fig. 2H. Disparities were observed in the HPLC outcomes during SQX degradation between the groups. Both groups identified the intermediate product 2-AQ at 3.410 min. However, a novel product was detected at 2.963 min by group AJ, differing from the degradation products identified in group AC at 5.473 min and 2.227 min (standard HHQ). Notable color changes in the medium during the degradation process indicated distinct functional roles of strains HAEJ-1 and HC-1 in the co-culture with HA-1 for SQX degradation. Subsequent experiments investigated the involvement of HC-1 and HAEJ-1 in degrading SQX and its products 2-AQ and HHQ. HAEJ-1 was able to degrade HHQ within 4 days, whereas HC-1 lacked this capability (Fig. S5A and S5B). When both HC-1 and HAEJ-1 were employed for SQX degradation, only HC-1 produced the N_4_-acetyl SQX degradation product at 5.473 min (Fig. S5C). Moreover, HAEJ-1 demonstrated the capacity to further degrade 2-AQ, unlike HC-1 (Fig. S5D). These findings suggest that HC-1 and HAEJ-1 not only facilitated the growth of HA-1 but also participated in the degradation of SQX and its degradation products. The outcomes from combining HA-1 with HC-1 or HAEJ-1 individually indicated that HA-1 alone did not degrade SQX in MSM medium. However, the intracellular supernatant addition of HC-1 or HAEJ-1 promoted SQX degradation by HA-1, albeit less effectively than the direct addition of strains HC-1 or HAEJ-1 (Fig. 2I and K). Additionally, when HA-1 was combined with HC-1 or HAEJ-1 in varying ratios, the degradation rate of SQX increased with a higher HA-1 ratio, underscoring the significant role of HA-1 as the primary degrading strain in the process (Fig. 2J and L).

### Mechanism of consortium ACJ co-culture

Genome sequencing of strains HA-1, HC-1, and HAEJ-1 was successfully completed, marking the documentation of the complete genome of a challenging-to-culture bacterium within *Leucobacter* sp. The genomic structures of these strains are visually represented in Fig. S6A, with details provided in Table S1. Analysis of ARGs revealed resistance to multiple antibiotic classes in these strains (Fig. S6B, S6D and S6F), with further details available in Table S2. Comparative analysis with previous research and the genomic data indicated that genes responsible for SA antibiotic degradation, specifically *sadA*, *sadB*, and *sadC*, were unique to strain HA-1. Strain HC-1 possessed acetylation genes (NATs), whereas HAEJ-1 did not. Notably, HAEJ-1 was primarily involved in the degradation of downstream products HHQ and 2-AQ, indicating that there were probably some specific functional genes involved in the degradation, but further research was needed.

**Fig 3:**
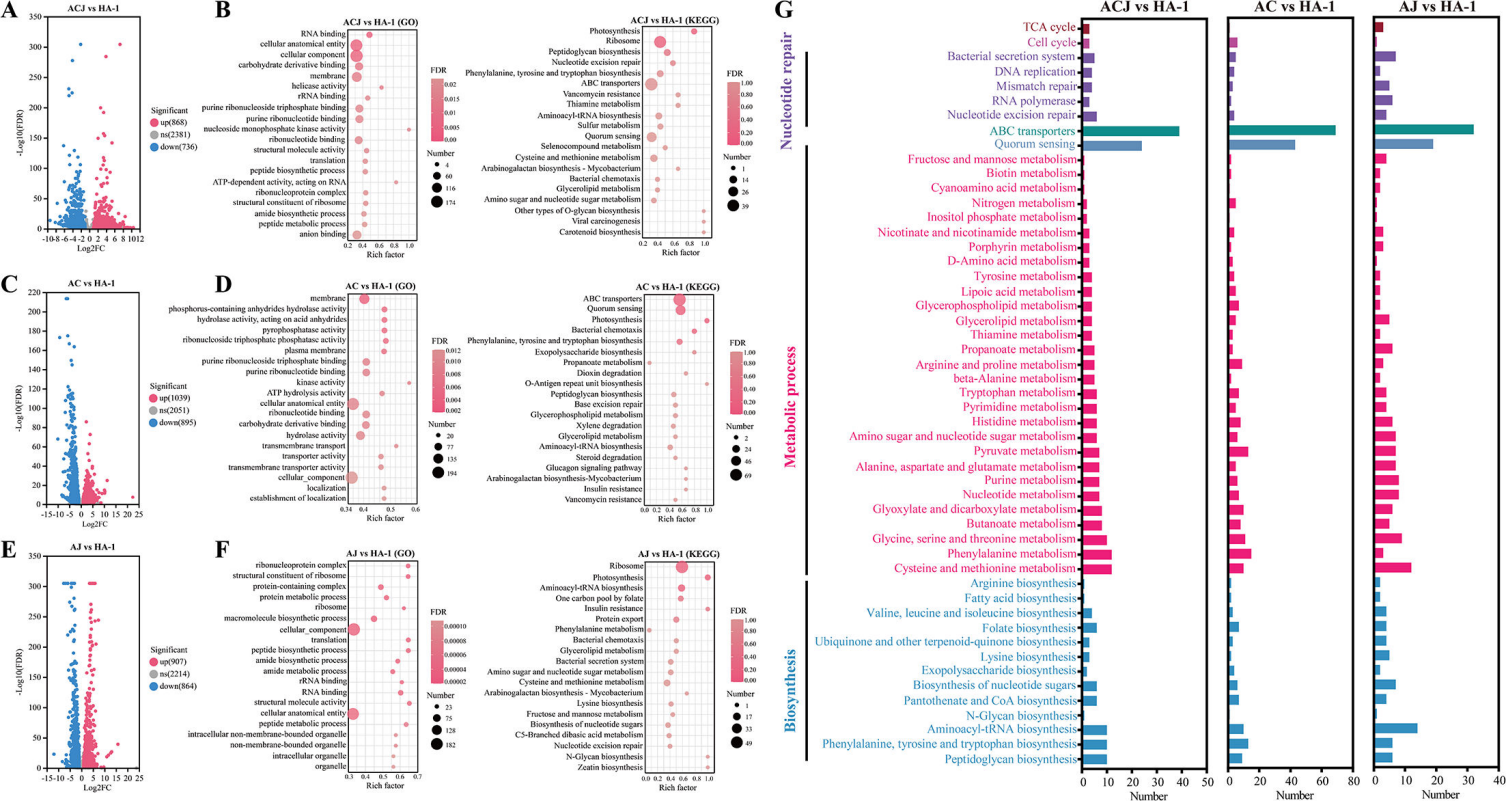
Transcriptome analysis of the response mechanism between strains in ACJ co-culture. Volcano plots of differential expression of HA-1 genes in co-culture vs monoculture ([A] ACJ vs HA-1, [C] AC vs HA-1, [E] AJ vs HA-1). Enrichment analysis of GO and KEGG functional categories of HA-1 in co-culture vs monoculture ([B] ACJ vs HA-1, [D] AC vs HA-1, [F] AJ vs HA-1). (G) Number and classification of upregulated genes in the enriched KEGG pathway of HA-1 in co-culture. Three replicates were used for transcriptomics analyses.

To explore the mechanisms of interaction between HA-1 co-cultured with HC-1 and HAEJ-1, transcriptomic and non-targeted metabolomics analyses were conducted for both monoculture and co-culture conditions. In the co-culture of the three strains, HA-1 exhibited 868 upregulated genes and 736 downregulated genes (group ACJ vs HA-1) (Fig. 3A), HC-1 showed 600 upregulated genes and 683 downregulated genes (group ACJ vs HC-1) (Fig. S7A), and HAEJ-1 displayed 465 upregulated genes and 215 downregulated genes (group ACJ vs HAEJ-1) (Fig. S7C). Co-culturing HA-1 with HC-1 led to 1,039 upregulated genes and 895 downregulated genes in HA-1 (group AC vs HA-1) (Fig. 3C), and co-culturing with HAEJ-1 resulted in 907 upregulated genes and 864 downregulated genes in HA-1 (group AJ vs HA-1) (Fig. 3E). Differentially expressed genes (DEGs) in the co-cultures were further analyzed using KEGG and GO functional category enrichment, focusing on the functional enrichment of upregulated genes in HA-1 across scenarios involving two and three strains, predominantly consisting of HA-1 (Fig. 3B, D and F). KEGG enrichment results indicated a significant response of HA-1’s upregulated genes to various metabolic pathways, notably in biosynthesis, metabolic processes, nucleotide repair, ABC transporters, quorum sensing, TCA cycle, and cell cycle pathways (Fig. 3G). These findings suggest that HA-1 exhibited multiple gene responses within the co-culture system, thereby enhancing its growth and degradation capabilities. In the co-culture system involving HC-1 and HAEJ-1, GO and KEGG enrichment analyses of upregulated genes in DEGs primarily highlighted biosynthetic and metabolic processes (Fig. S7B and S7D).

**Fig 4:**
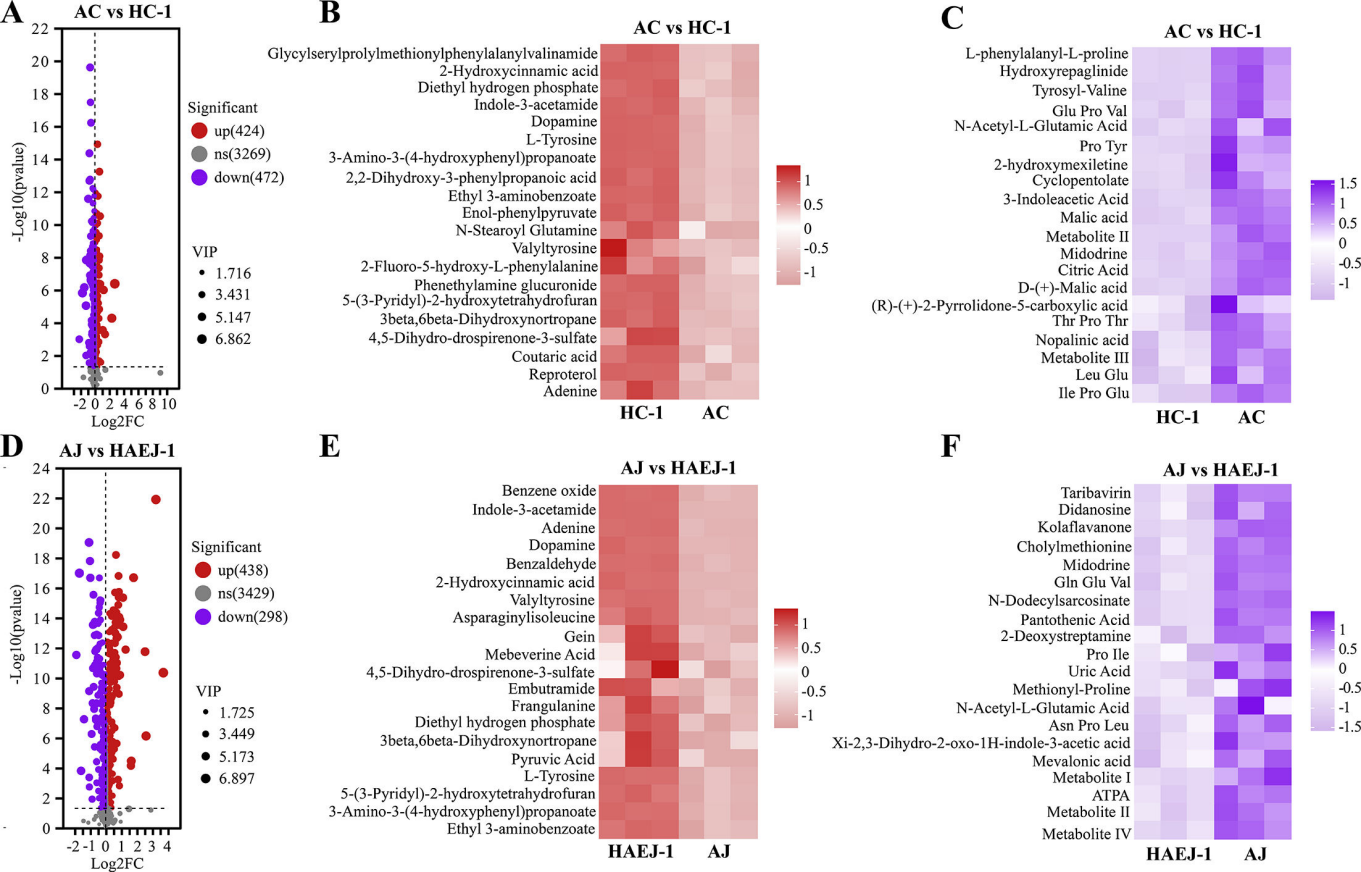
Metabolome analysis of growth factors utilized and metabolized during HA-1 growth. (A) Volcanic plots of intracellular differential metabolites of HC-1 with and without HA-1. (B) Top 20 metabolite abundance of HC-1 intracellular was used in the growth of HA-1. (C) Abundance of top 20 metabolites produced when HC-1 intracellular was used in the growth of HA-1. (D) Volcanic plots of intracellular differential metabolites of HAEJ-1 with and without HA-1. (E) Top 20 metabolite abundance of HAEJ-1 intracellular was used in the growth of HA-1. (F) Abundance of top 20 metabolites produced when HAEJ-1 intracellular was used in the growth of HA-1. Four replicates were used for metabolomics analyses.

Non-targeted metabolomics studies were conducted to investigate the substances utilized and metabolized by the HA-1 strain during its growth. The analysis focused on the changes in the medium’s composition when HA-1 was cultured with intracellular supernatant from HC-1 or HAEJ-1. A comparison between groups AC vs HC-1 and AJ vs HAEJ-1 revealed that 424 substances increased and 472 decreased in the former, while 438 substances increased and 298 decreased in the latter (Fig. 4A and D). Meanwhile, the top 100 metabolites with higher abundance in 1/10 LB medium were analyzed. It was found that these metabolites included amino acids, peptides, organic acids, purines, and their derivatives, which were highly likely to be utilized for the growth of strain HA-1 (Fig. S8). This indicated that the strain HA-1 utilized the metabolite components in the culture medium containing the intracellular supernatants of HC-1 or HAEJ-1, it was very likely that the strain HA-1 also metabolized new substances during its growth process. However, whether these substances could be effectively utilized by strains HAEJ-1 and HC-1 needs further study. Further analysis of the top 20 altered substances in different treatments showed that the substances utilized or metabolized by HA-1 in the presence of medium with intracellular supernatant from HC-1 or HAEJ-1 were distinct (Fig. 4B, C, E and F). However, all identified substances, categorized as amino acids, peptides and analogs, organic acids, organic heterocyclic compounds, benzene compounds, phenylpropanes, polyketide compounds, alkaloids, and their derivatives, were consistent with the findings from transcriptomic and targeted metabolomics analyses, demonstrating that the substances utilized and metabolized by HA-1 in the co-culture system aligned with the gene transcription results of the three strains.

**Fig 5:**
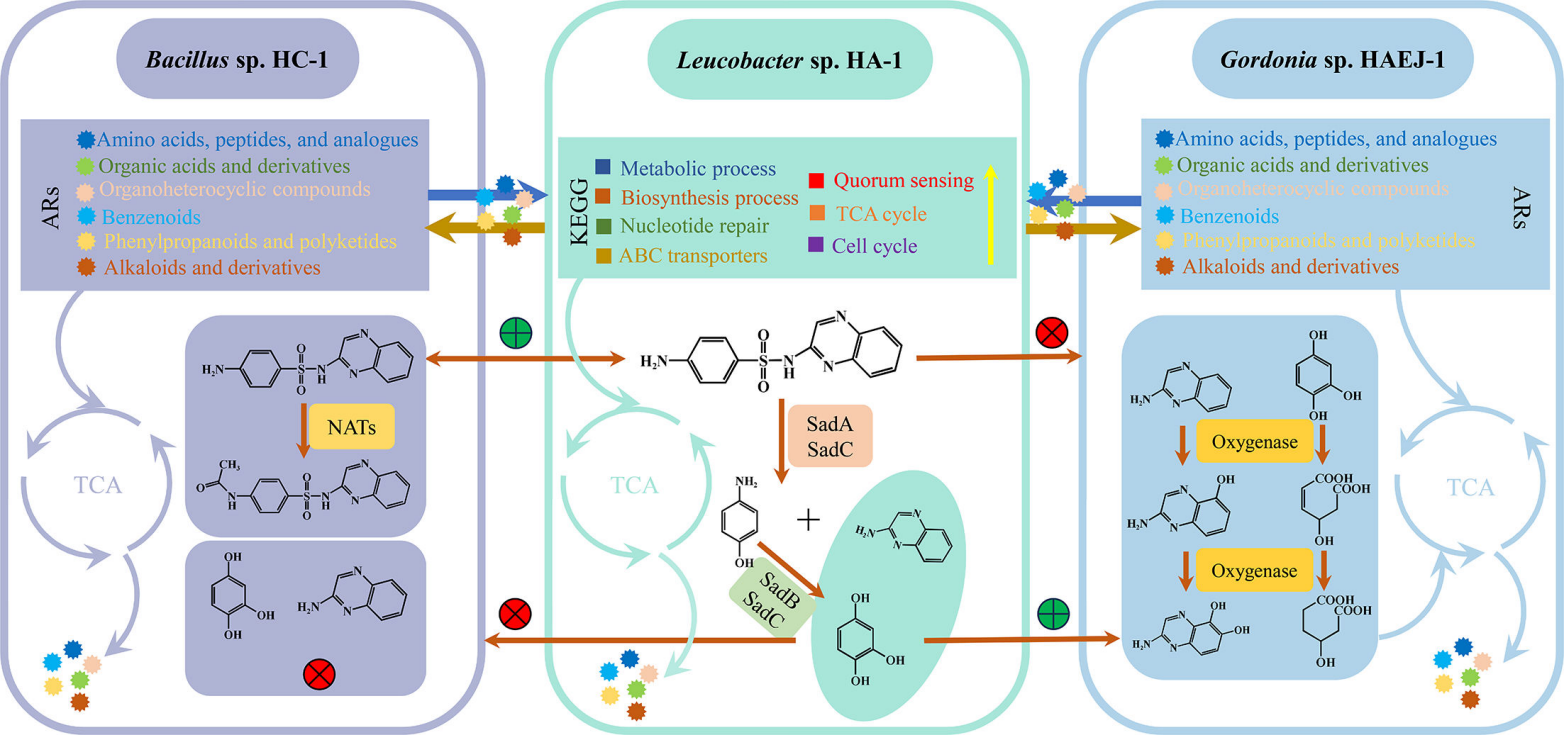
Schematic representation of the interaction between HA-1, HC-1, and HAEJ-1 during degradation of SQX and co-culture. SQX can be degraded synergically among the members of the consortium ACJ. SadABC in HA-1 degrades SQX to HHQ and 2-AQ, which may be further degraded by the oxygenase in HAEJ, but not HC-1, but there are some of the acetylase NATs in HC-1 that transform SQX to N_4_-acetyl SQX. In ACJ co-culture, each strain not only has the response of many important metabolic pathways related genes but also the mutual transfer and cross-feeding of key ARs. HHQ represents trihydroxybenzene. 2-AQ represents 2-aminoquinoxaline. ARs represent the auxotrophic requirements. The green circle means degradable, and the red circle means non-degradable. The colored pellets represent the essential ARs provided by strains HC-1 or HAEJ-1 to strain HA-1.

Co-culture experiments demonstrated that when strains HC-1 or HAEJ-1 were co-cultured with strain HA-1 in 1/10 LB medium with or without 50 mg/L SQX, there was an increase in biomass of strains HC-1 or HAEJ-1 compared with that of a single culture. At 96 h, the biomass of HC-1 was found to be 3.7 × 10^8^ CFU/mL when HC-1 and HA-1 were co-cultured in 1/10 LB medium containing 50 mg/L SQX, while in the medium without SQX was 3.1 × 10^8^ CFU/mL. When cultured alone, the biomass of HC-1 was only 2.85 × 10^8^ CFU/mL (Fig. S9A). A similar trend was observed in the co-culture of HAEJ-1 and HA-1 in 1/10 LB medium containing 50 mg/L SQX, where the biomass of HAEJ-1 reached 3.6 × 10^8^ CFU/mL. The biomass of HAEJ-1 cultured without SQX was 2.55 × 10^8^ CFU/mL, while that of HAEJ-1 cultured alone was only 2.1 × 10^8^ CFU/mL (Fig. S9B). Through the integration of genomes, transcriptomes, metabolomes, and SQX metabolic pathways, a preliminary investigation into the synergistic degradation of SQX by *Leucobacter* sp. HA-1, *Bacillus* sp. HC-1, and *Gordonia* sp. HAEJ-1 was conducted. Our findings, illustrated in Fig. 5 , demonstrated that co-cultivation of HA-1 with HC-1 or HAEJ-1 triggers a genetic response in each strain, facilitating the provision of ARs through cross-feeding. Additionally, a collaborative approach involving division of labor was employed for SQX degradation, with the resulting degradation products being metabolized to contribute to the TCA cycle, thereby sustaining the growth and catabolic activities of ACJ.

### Degradation consortium ACJ reduced the toxicity effect of SQX

**Fig 6:**
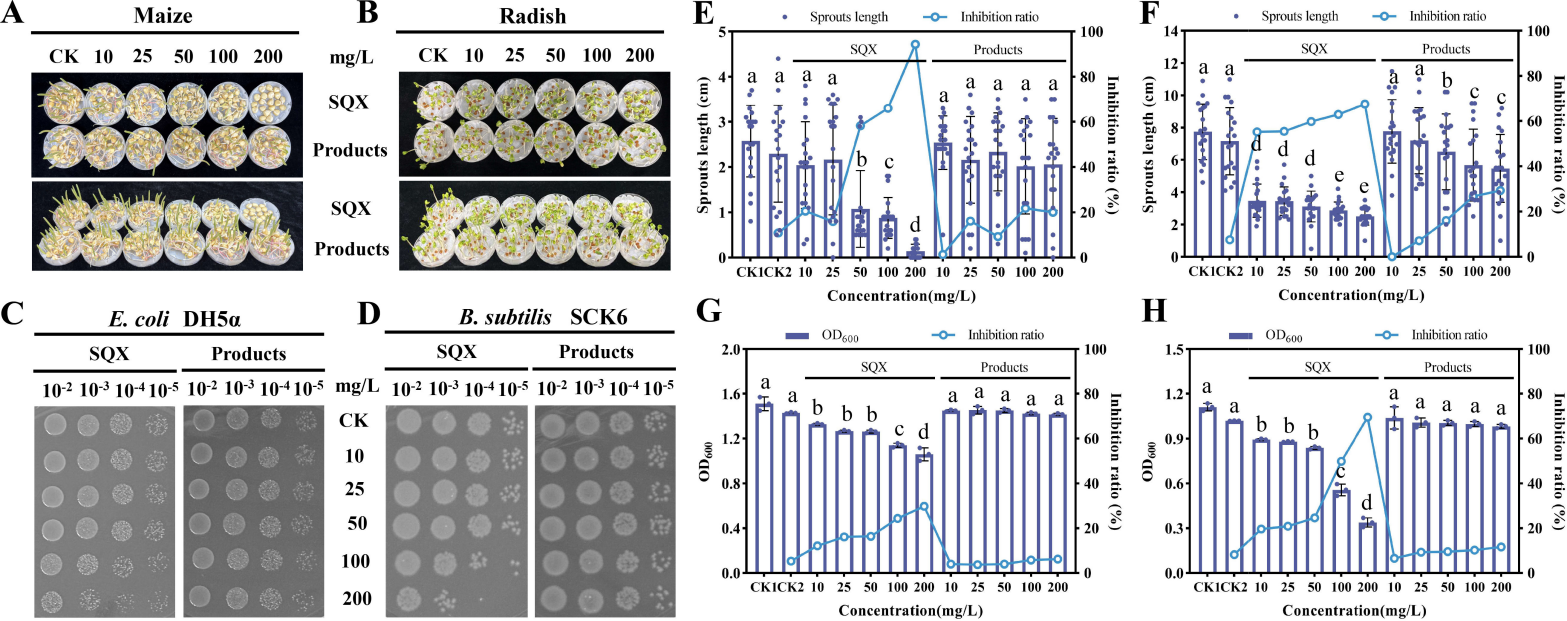
Toxicity comparison of SQX and its products to seeds and bacteria. Toxicity test of SQX and its degradation products on maize seeds (**A**) and radish seeds (**B**), 20 seeds were placed in each petri dish. Toxicity test of SQX and its degradation products on *E. coli* DH5α (**C**) and *B. subtilis* SCK6 (**D**). Sprout length and inhibition rate of SQX and degradation products on seed germination of 20 maize seeds (**E**) or radish seeds (**F**). OD_600_ and inhibition rate of SQX and degradation products on the growth of *E. coli* DH5α (**G**) and *B. subtilis* SCK6 (**H**). The error bar represents three repetitions. Different letters indicate significance tests as determined by one-way ANOVA (*P* < 0.05).

Studies have demonstrated that SAs inhibit the growth of microorganisms and plants (21, 40). Consequently, it is crucial to evaluate the toxic effects of SAs on organisms. For this assessment, seeds of maize and radish, along with the gram-negative bacterium *E. coli* DH5α and the gram-positive bacterium *B. subtilis* SCK6, were selected. The presence of SQX notably hindered the germination of maize and radish seeds. However, exposure to the degradation supernatant of SQX significantly reduced this toxicity. Specifically, SQX at a concentration of 50 mg/L markedly impeded maize seed germination (Fig. 6A). In the experiment, maize and radish seeds were treated with buffered solutions containing various concentrations of SQX and its degradation supernatant. The sprout lengths of 20 seeds from each species were measured after several days of germination under controlled light conditions. Inhibition rates of seed germination were 10.87%, 20.58%, 15.92%, 58.25%, 66.02%, and 94.37% at SQX concentrations of 0, 10, 25, 50, 100, and 200 mg/L, respectively, compared to the control group germinated solely with water. In contrast, inhibition rates for the degradation supernatant at the same concentrations were 1.36%, 16.12%, 9.32%, 21.75%, and 20.00%, respectively (Fig. 6E). Notably, radish seeds showed higher sensitivity to SQX than maize, with even low concentrations significantly impeding germination (Fig. 6B). When radish seeds were exposed to the same concentrations of SQX and its degradation supernatant as maize seeds, inhibition rates of 7.56%, 55.17%, 55.49%, 59.75%, 62.92%, and 67.51% were recorded for SQX, while the degradation products exhibited inhibition rates of 0, 7.11%, 16.02%, 26.68%, and 29.33%, respectively (Fig. 6F). Cultivation of DH5α and SCK6 bacteria in media containing various concentrations of SQX or its degradation supernatant showed that only high concentrations of SQX effectively inhibited bacterial growth (Fig. 6C and D). Measurement of OD_600_ values for DH5α and SCK6 under different treatment conditions indicated an increase in growth inhibition rates with rising SQX concentrations. Specifically, DH5α and SCK6 exhibited OD_600_ values of 1.51 and 1.11, respectively, in the absence of SQX, but at SQX concentrations of 100 and 200 mg/L, DH5α displayed inhibition rates of 24.4% and 29.77% (with OD_600_ values of 1.14 and 1.06), while SCK6 exhibited inhibition rates of 49.91% and 69.53% (with OD_600_ values of 0.56 and 0.34), respectively (Fig. 6G and H).

## DISCUSSION

Several prior studies have indicated that microbial consortium has the capability to degrade SAs more efficiently than individual bacteria, with potential for mineralization as well (12, 41, 42). Variations in the degradation efficiency of different types of SAs within the same consortium have been observed, potentially attributed to the diverse heterocyclic structures present (17, 43, 44). This aligned with findings from the degradation assessment of six kinds of SAs (SQX, SMZ, SMX, ST, SP, and SIZ) by ACJ in the current investigation (Fig. S2A)), an ACJ bacterial suspension with an OD_600_ of 0.5 could degrade SAs with a concentration of up to 50 mg/L to undetectable levels within 7 days, and the degradation efficiency was more advantageous than other studies (45–47). We assessed the potential toxicity of SAs and their products on plant seed germination and microorganisms. Our findings demonstrate that the degradation of SAs by consortium ACJ significantly decreased the inhibition rate of seed germination and bacterial growth (Fig. 6). The resulting degradation products were environmentally friendly, with reduced toxicity compared to the parent compound (48–50). Furthermore, a variety of SAs composed of distinct heterocycles may follow different metabolic pathways, while SMX and SMZ have been extensively studied, SQX, despite its significant consumption and environmental impact, lacks comprehensive metabolic pathway documentation (17, 51). This study primarily focused on the degradation of SQX by ACJ, it found that the degradation of SQX by ACJ was a new metabolic pathway, especially for the further metabolism of the end product 2-AQ (Fig. 1G). We have discovered that strain HAEJ-1 has the ability to further catabolize 2-AQ, resulting in the production of new mono- or dual-oxygenated products. This finding represents a valuable addition to the metabolic pathway involved in the microbial degradation of SQX. Many studies have shown that there is always a continuous accumulation of end products in the degradation of SAs (17, 18). The results of this study will provide new insights into the further metabolism of SAs. Within the microbial consortium, various members assume leading and supporting roles in the degradation process, with supporting roles potentially lacking degradation functions (52). The consortium comprising HA-1, HC-1, and HAEJ-1 was established for SAs degradation, the interesting phenomenon is that HA-1 alone cannot play a role in degradation, and the addition of HC-1 and HAEJ-1 not only initiates the degradation of SAs but also participates in the further metabolism of downstream products, indicating that there is a co-degradation relationship among the bacterial consortium ACJ.

In the reported SAs-degrading consortium, the bacteria belonging to the genus *Leucobacter* are known for their challenging culturing requirements, often necessitating the presence of auxiliary bacteria for growth and degradation processes, as documented in previous research (11). Despite this, an effective culture method for these strains has yet to be identified. This current investigation revealed that the HA-1 strain exhibited the ability to utilize both extracellular and intracellular components of HC-1 or HAEJ-1 for its proliferation. Consequently, HA-1 was successfully isolated and cultured independently, leading to the complete sequencing of its genome (Fig. 2A and B and S6A). Notably, three strains harbor numerous antibiotic resistance genes, notably including the SAs resistance gene *sul* and efflux pump genes, which likely contribute to their survival in environments contaminated with SAs over extended periods (53, 54). Prior studies have established that the degradation of SAs is initiated by two FMNH2-dependent monooxygenases, SadA and SadB, along with a flavin reductase SadC, representing the sole functional gene associated with sulfanilamide degradation (53, 55). The presence of SadABC in the HA-1 genome further supports the assertion that this strain serves as the primary degrading bacterium in the ACJ. Similarly, genomic analyses of HC-1 and HAEJ-1 revealed the presence of the sulfanilamide acetylase gene NATs in HC-1 (56, 57), the HHQ ring cleavage dioxygenase gene in HAEJ-1 (58, 59), and candidate functional genes for degrading 2-AQ in HAEJ-1. It is noteworthy that none of these genes exhibited homologous counterparts in other member strains, thereby emphasizing the distinct functional specialization of ACJ members in the SAs degradation process.

Metabolite cross-feeding serves as the foundation for interspecies synergies, as highlighted in recent studies (24, 27). In the realm of degradation, beyond the shared utilization of pollutant intermediate metabolites, this concept encompasses the provision of nutrients by strains, referred to as ARs in the present investigation. Research on bacterial co-cultures has demonstrated that amino acids, vitamins, sugars, NH_4_^+^, and similar substances can function as cross-feeding agents within bacterial consortiums, sustaining the growth and functionality of the strains involved (30, 32, 60, 61). This study specifically delved into the genetic responses of strain HA-1 when co-cultured with other strains, along with the particular substances involved in cross-feeding. The intricate molecular and metabolic mechanisms underlying microbial consortium interactions can be elucidated through transcriptomics and metabolomics analyses (62–64). Transcriptomics was employed to investigate the molecular mechanisms at play in the co-culture of HA-1, HC-1, and HAEJ-1, revealing differential gene expression patterns compared to individual cultures. Notably, for HC-1 and HAEJ-1, which exhibited normal growth in LB medium, upregulated genes primarily pertained to substance biosynthesis and metabolism when co-cultured with HA-1. This observation suggested that HC-1 and HAEJ-1 supplied ARs to HA-1 during co-cultivation. Gene enrichment analyses indicated that the upregulated genes in strain HA-1 were predominantly associated with biosynthesis, metabolic processes, nucleotide repair, ABC transporters, quorum sensing, the TCA cycle, and the cell cycle. The TCA cycle and ABC transporters represent key pathways that provide energy to the bacteria (65), while nucleotide repair and the cell cycle are crucial for rectifying cellular damage and maintaining physiological functions (57). Quorum sensing facilitates interspecies communication in microbial co-cultures, playing a pivotal role in coordinating cell signaling and gene regulation (66, 67). These findings underscored that the challenging-to-culture strain HA-1 could induce the expression of numerous genes linked to physiological metabolism, thereby enhancing its own growth when co-cultured with other strains.

In the context of organic pollutant degradation, the exchange of metabolites among microbial communities, known as cross-feeding, plays a significant role in interspecific interactions (68, 69). Experimental findings from purification and culture studies of strain HA-1 indicated its ability to grow in LB medium with the intracellular supernatant from strains HC-1 or HAEJ-1, with enhanced growth observed as intracellular supernatant content increased (Fig. 2A through 2F). Metabolomics was employed to identify the specific metabolic substances utilized by HA-1. When intracellular components of HC-1 or HAEJ-1 were introduced for HA-1 culture, a reduction in the abundance levels of various substances compared to the control was observed, confirming HA-1’s utilization of LB medium with the intracellular supernatant from strains HC-1 or HAEJ-1 for growth, consistent with experimental outcomes. These utilized substances primarily included amino acids, peptides, organic acids, organic heterocyclic compounds, benzene compounds, phenylpropanes, polyketide compounds, alkaloids, and their derivatives. The findings suggested that HA-1 may not rely on a singular type or class of ARs. Previous research has demonstrated the use of zincmethylphyrins and coproporphyrins as novel ARs for culturing previously uncultured *Leucobacter* sp. ASN212 (35). However, attempts to culture HA-1 by supplementing the medium with these substances were unsuccessful. Furthermore, during HA-1 growth in 1/10 LB medium with the strains HC-1 or HAEJ-1 intracellular supernatant, resulting in the secretion of various amino acids and organic acids. This finding suggested a potential reciprocal utilization of these substances by HC-1 or HAEJ-1 to support their own growth. Nevertheless, further research was required to ascertain whether these substances could be utilized effectively by strains HAEJ-1 and HC-1 (Fig. 4C and F and S9). To summarize, a mutually beneficial relationship was identified in the consortium ACJ.

In summary, all species HA-1, HC-1, and HAEJ-1 within the consortium ACJ exhibited synergistic degradation of SAs. A complex mutually beneficial relationship was identified among these three strains. During co-culture degradation, multiple metabolic pathway genes were activated, along with cross-feeding and degradation of metabolites. The potential toxicity and environmental risks of SAs biodegradation were comprehensively evaluated. This study contributed valuable resources of SAs degrading strains, elucidated the metabolic pathways of SAs, the division of labor among each strain in degradation, and analyzed the interspecific interaction mechanism of ACJ using transcriptome and metabolome analyses.

## Data Availability

The genome and transcriptome raw data in this study are available from the National Center for Biotechnology Information (BioProject PRJNA1128373, PRJNA1128426, PRJNA1128391, and PRJNA1129438). The metabolomics raw data of GC-MS have been deposited in Metabolights (MTBLS11473) (70).

## SUPPLEMENTAL MATERIAL

Supplemental material aem.00590-25-s0001.pdf Fig. S1 to S9; Tables S1 and S2.

ASM does not own the copyrights to Supplemental Material that may be linked to, or accessed through, an article. The authors have granted ASM a non-exclusive, world-wide license to publish the Supplemental Material files. Please contact the corresponding author directly for reuse.
