# Supplementary material for: Cross-feeding and co-degradation within a bacterial consortium dominated by challenging-to-culture Leucobacter sp. HA-1 enhances sulfonamide degradation
Source: Appl Environ Microbiol. 2025 Jun 24;91(7):e00590-25. doi: 10.1128/aem.00590-25 (PMC12285269; doi:10.1128/aem.00590-25)
Supplement: Supplemental material — Fig. S1 to S9; Tables S1 and S2. [file aem.00590-25-s0001.pdf]

Supplementary material

Cross-feeding and co-degradation within a bacterial consortium dominated by challenging-to-culture *Leucobacter* sp. HA-1 enhances sulfonamides degradation

Guoqiang Zhao[1], Houyu Yu[1], Juanjuan Wang[1], Bo Jiang[3], Fangya Zhong[1], Rui Zhang[1], Tianzhi Jiang[2], Mo Yang[2], Hui Wang[2], Xing Huang[1, #]

[1] Department of Microbiology, College of Life Sciences, Nanjing Agricultural University, Nanjing, Jiangsu 210095, PR China.

[2] State Key Laboratory of Biocatalysis and Enzyme Engineering, School of Life Sciences, Hubei University, Wuhan, Hubei 430062, China.

[3] Jingzhou Municipal Ecological Environment Information and Assessment Center, Jingzhou, Hubei 434000, PR China.

# Corresponding author. Tel/fax: +86 2584395326

E-mail address: huangxing@njau.edu.cn (X. Huang)

Number of Pages: 15

Number of Figures: 9

Number of Tables: 2

TABLE AND FIGURE CAPTIONS

Fig. S1. Phylogenetic tree analysis of bacterial consortium ACJ based on 16S rRNA. (A) HA-1, (B) HC-1 and (C) HAEJ-1.

Fig. S2. SAs degradation characteristics of consortium ACJ. A. Degradation of multiple SAs by consortium ACJ. Degradation of SQX, SMZ and SMX by ACJ under different temperature (B) and pH (C) conditions. Error bars represent the standard deviations of three replicates.

Fig. S3. Identification of SQX, SMZ and SMX metabolic intermediates by HPLC-MS/MS. The exclusive metabolites of SQX (RT 1.212 min, m/z 299.0594) include metabolites a (RT 2.101 min, m/z 341.0691), b (RT 3.910 min, m/z 144.0561), c (RT 3.253 min, m/z 160.0506), and d (RT 1,728 min, m/z 176.0456). The exclusive metabolites of SMX (RT 4.771 min, m/z 254.0594) include metabolites e (RT 6.152 min, m/z 296.0702) and f (RT 1.068 min, m/z 99.0556). The exclusive metabolites of SMZ (RT 4.224 min, m/z 279.0915) include metabolites g (RT 5.224 min, m/z 321.1020) and h (RT 1.564 min, m/z 124.0873). The common metabolites of SQX, SMZ and SMX include metabolites i (RT 1.654 min, m/z 110.0602), j (RT 1.146 min, m/z 111.0440), k (RT 0.784 min, m/z 125.0353), l (RT 0.587 min, m/z 159.0291), and m (RT 3.402 min, m/z 161.0528). Each mass spectrum result contains a primary mass spectrum (bottom left) and a secondary mass spectrum (top right). The chemical structure formulas in the figure are inferred from the HPLC-MS/MS results.

Fig. S4. Status of SQX degraded by HA-1 alone in LB medium added intracellular supernatant of HAEJ-1 and HC-1. The number represents the volume of intracellular supernatant added, and each vial contains the same volume of solution. The color of the degradation reaction solution changed from yellow to dark brown with the increase in the amount of intracellular supernatant added.

Fig. S5. The role and division of strains HC-1 and HAEJ-1 in the degradation of SQX. A. The degradation of HHQ by HAEJ-1 (A) and HC-1 (B) was determined by HPLC. The degradation of SQX (C) and 2-AQ (D) by HAEJ-1 and HC-1 was determined by HPLC. HHQ represents trihydroxybenzene. 2-AQ represents 2-aminoquinoxaline.

The chemical structure formula represents the compound detected by HPLC, and the time indicates the specific peak time of the compound in HPLC.

Fig. S6. The genome mapping and antibiotic resistance gene analysis of HA-1, HC-1 and HAEJ-1. A. The genome mapping of strain HA-1. B. The antibiotic resistance gene analysis of HA-1, there are six sulfonamide antibiotic resistance genes. C. The genome mapping of strain HC-1. D. The antibiotic resistance gene analysis of strain HC-1, there are two sulfonamide antibiotic resistance genes in HC-1. E. The genome mapping of strain HAEJ-1. F. The antibiotic resistance gene analysis of HAEJ-1, there are six sulfonamide antibiotic resistance genes.

Fig. S7. Transcriptome analysis of the response mechanism between strains in ACJ co-culture. A. Volcano plots of differential expression of HC-1 genes in co-culture versus monoculture (ACJ vs HC-1). B. Enrichment analysis of GO and KEGG functional categories of HC-1 in co-culture versus monoculture (ACJ vs HC-1). C. Volcano plots of differential expression of HAEJ-1 genes in co-culture versus monoculture (ACJ vs HAEJ-1). D. Enrichment analysis of GO and KEGG functional categories of HAEJ-1 in co-culture versus monoculture (ACJ vs HAEJ-1).

Fig. S8. Metabolome analysis of 1/10 LB medium. The top 100 metabolites of 1/10 LB medium with relatively high abundance were shown on the left. The relative abundance legend was shown on the right.

Fig. S9. Growth of strains HC-1 and HAEJ-1 under the conditions of single culture and co-culture. Biomass of (A) strain HC-1 or (B) strain HAEJ-1 cultured alone and co-cultured with strain HA-1 in 1/10 LB medium with or without SQX. Error bars represent the standard deviations of three replicates.

Table S1. The genome-related information of HA-1, HC-1 and HAEJ-1.

Table S2. The antibiotic resistance gene information of HA-1, HC-1 and HAEJ-1.

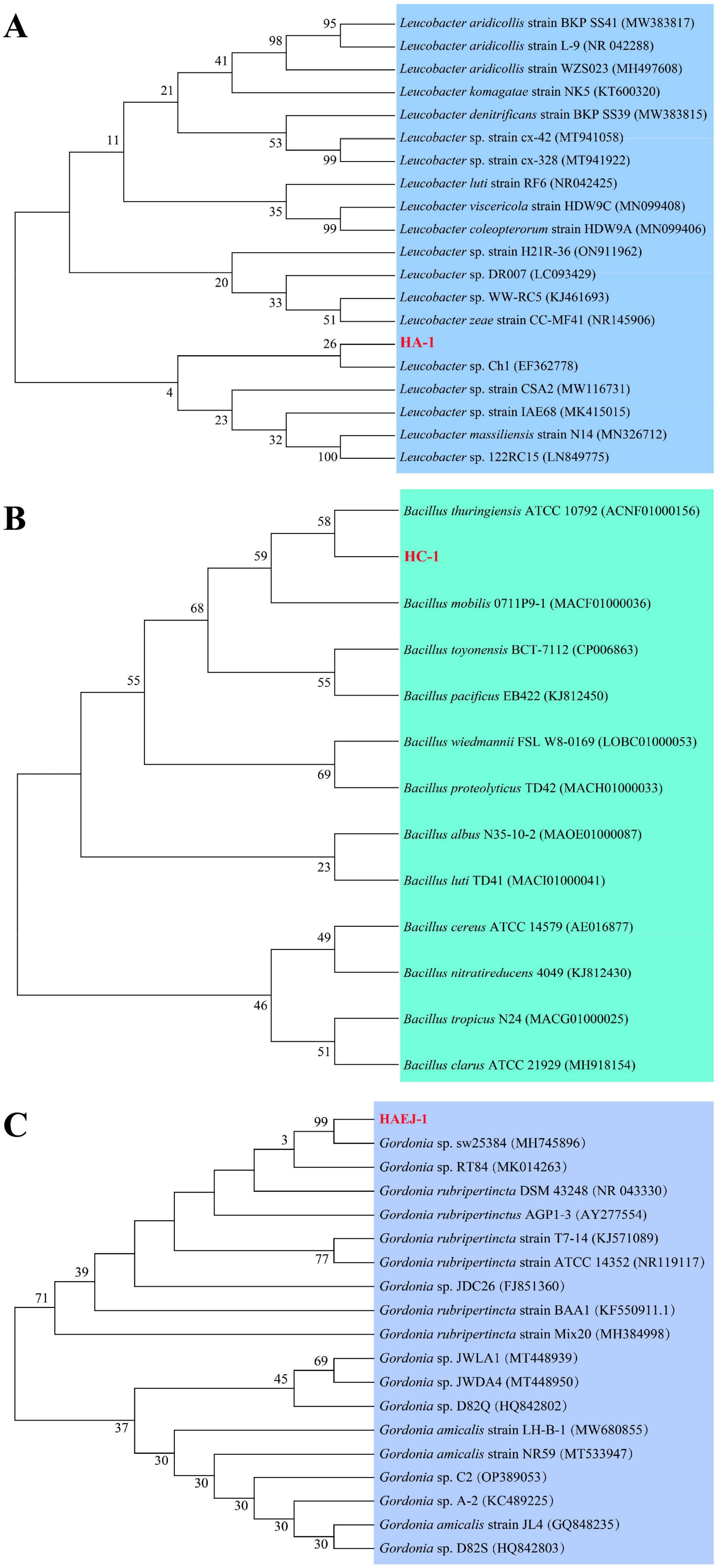

Fig. S1. Phylogenetic tree analysis of bacterial consortium ACJ based on 16S rRNA. (A) HA-1, (B) HC-1 and (C) HAEJ-1.

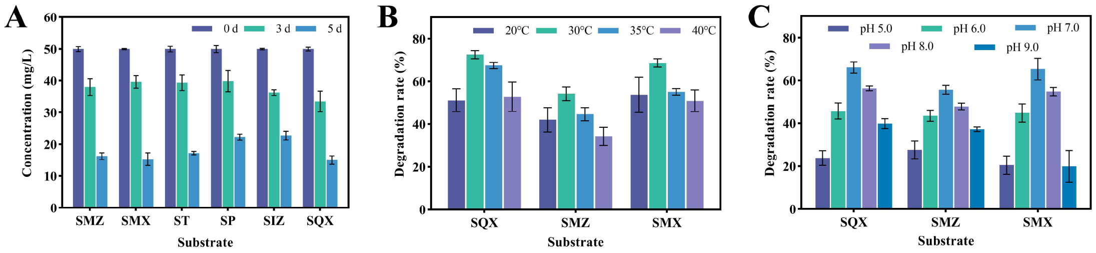

Fig. S2. SAs degradation characteristics of consortium ACJ. A. Degradation of multiple SAs by consortium ACJ. Degradation of SQX, SMZ and SMX by ACJ under different temperature (B) and pH (C) conditions. Error bars represent the standard deviations of three replicates.

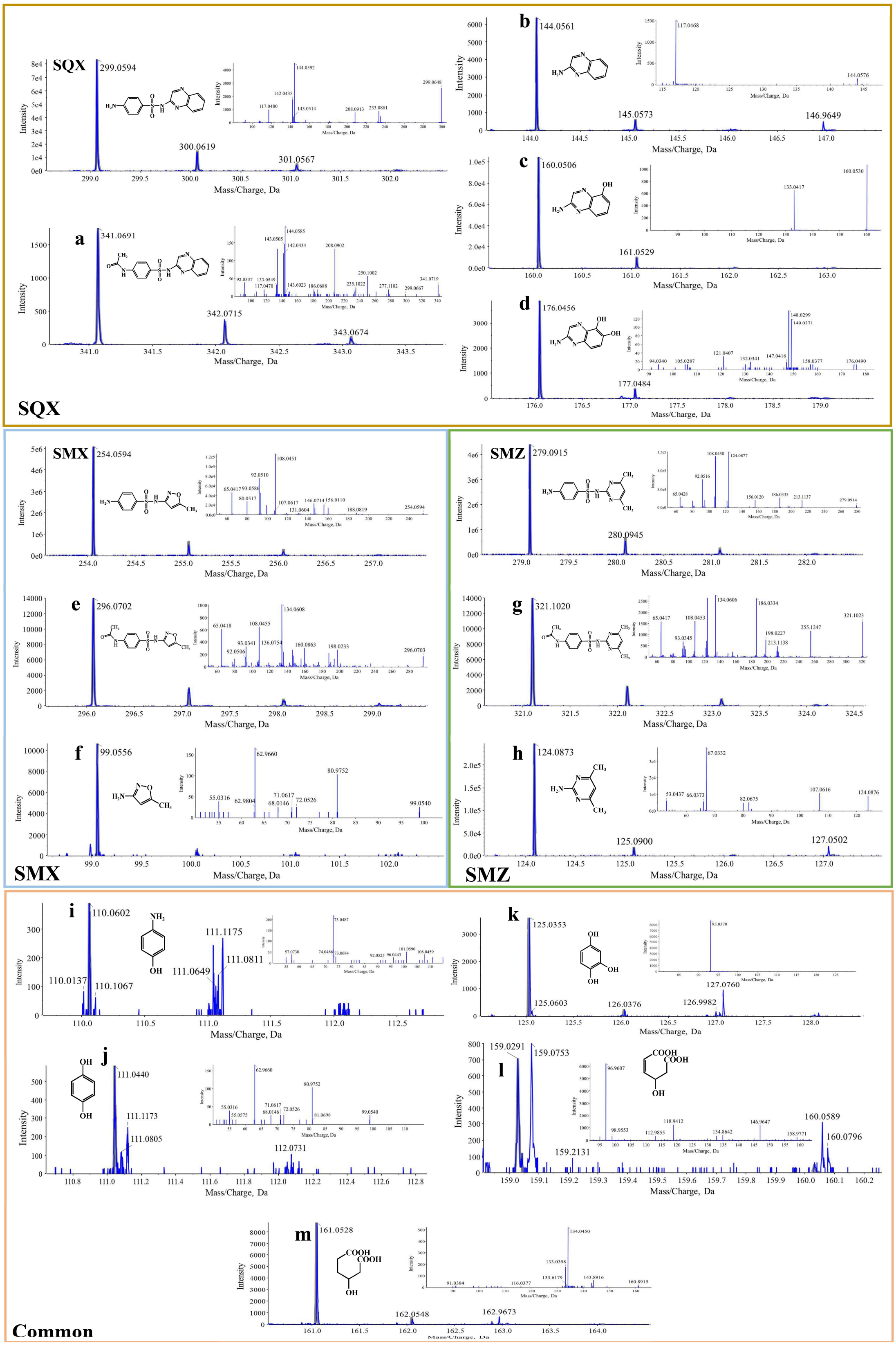

Fig. S3. Identification of SQX, SMZ and SMX metabolic intermediates by HPLC-MS/MS. The exclusive metabolites of SQX (RT 1.212 min, m/z 299.0594) include metabolites a (RT 2.101 min, m/z 341.0691), b (RT 3.910 min, m/z 144.0561), c (RT

3.253 min, m/z 160.0506), and d (RT 1,728 min, m/z 176.0456). The exclusive metabolites of SMX (RT 4.771 min, m/z 254.0594) include metabolites e (RT 6.152 min, m/z 296.0702) and f (RT 1.068 min, m/z 99.0556). The exclusive metabolites of SMZ (RT 4.224 min, m/z 279.0915) include metabolites g (RT 5.224 min, m/z 321.1020) and h (RT 1.564 min, m/z 124.0873). The common metabolites of SQX, SMZ and SMX include metabolites i (RT 1.654 min, m/z 110.0602), j (RT 1.146 min, m/z 111.0440), k (RT 0.784 min, m/z 125.0353), l (RT 0.587 min, m/z 159.0291), and m (RT 3.402 min, m/z 161.0528). Each mass spectrum result contains a primary mass spectrum (bottom left) and a secondary mass spectrum (top right). The chemical structure formulas in the figure are inferred from the HPLC-MS/MS results.

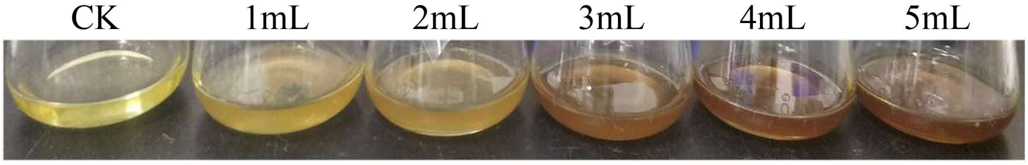

Fig. S4. Status of SQX degraded by HA-1 alone in LB medium added intracellular supernatant of HAEJ-1 and HC-1. The number represents the volume of intracellular supernatant added, and each vial contains the same volume of solution. The color of the degradation reaction solution changed from yellow to dark brown with the increase in the amount of intracellular supernatant added.

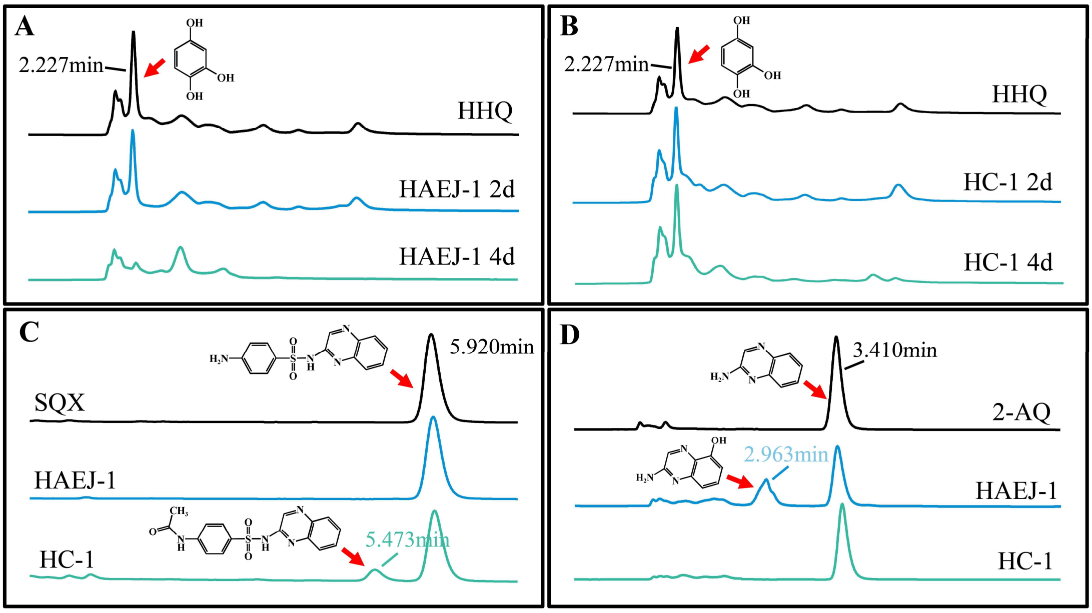

Fig. S5. The role and division of strains HC-1 and HAEJ-1 in the degradation of SQX. A. The degradation of HHQ by HAEJ-1 (A) and HC-1 (B) was determined by HPLC. The degradation of SQX (C) and 2-AQ (D) by HAEJ-1 and HC-1 was determined by HPLC. HHQ represents trihydroxybenzene. 2-AQ represents 2-aminoquinoxaline. The chemical structure formula represents the compound detected by HPLC, and the time indicates the specific peak time of the compound in HPLC.

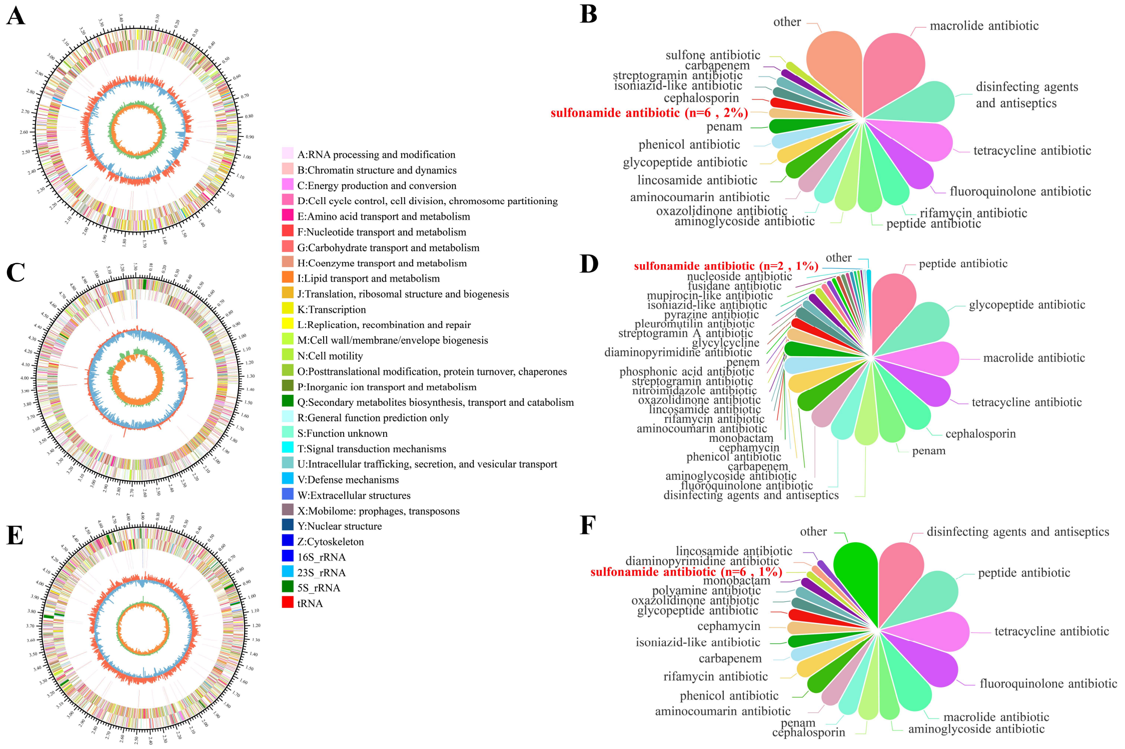

Fig. S6. The genome mapping and antibiotic resistance gene analysis of HA-1, HC-1 and HAEJ-1. A. The genome mapping of strain HA-1. B. The antibiotic resistance gene analysis of HA-1, there are six sulfonamide antibiotic resistance genes. C. The genome mapping of strain HC-1. D. The antibiotic resistance gene analysis of strain HC-1, there are two sulfonamide antibiotic resistance genes in HC-1. E. The genome mapping of strain HAEJ-1. F. The antibiotic resistance gene analysis of HAEJ-1, there are six sulfonamide antibiotic resistance genes.

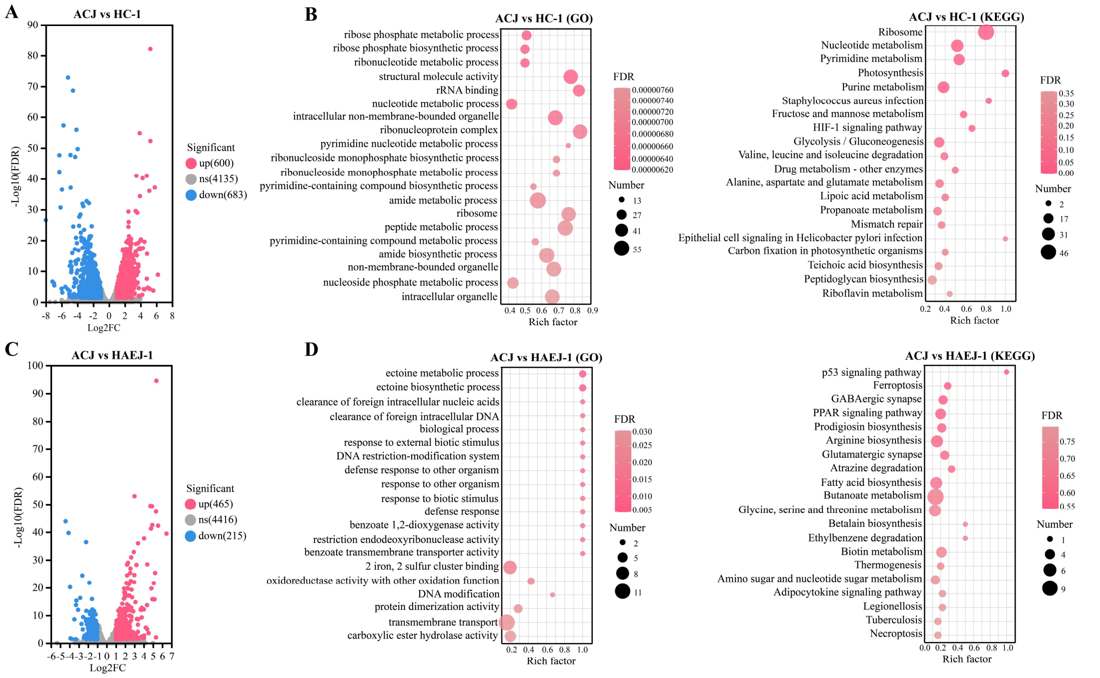

Fig. S7. Transcriptome analysis of the response mechanism between strains in ACJ co-culture. A. Volcano plots of differential expression of HC-1 genes in co-culture versus monoculture (ACJ vs HC-1). B. Enrichment analysis of GO and KEGG functional categories of HC-1 in co-culture versus monoculture (ACJ vs HC-1). C. Volcano plots of differential expression of HAEJ-1 genes in co-culture versus monoculture (ACJ vs HAEJ-1). D. Enrichment analysis of GO and KEGG functional categories of HAEJ-1 in co-culture versus monoculture (ACJ vs HAEJ-1).

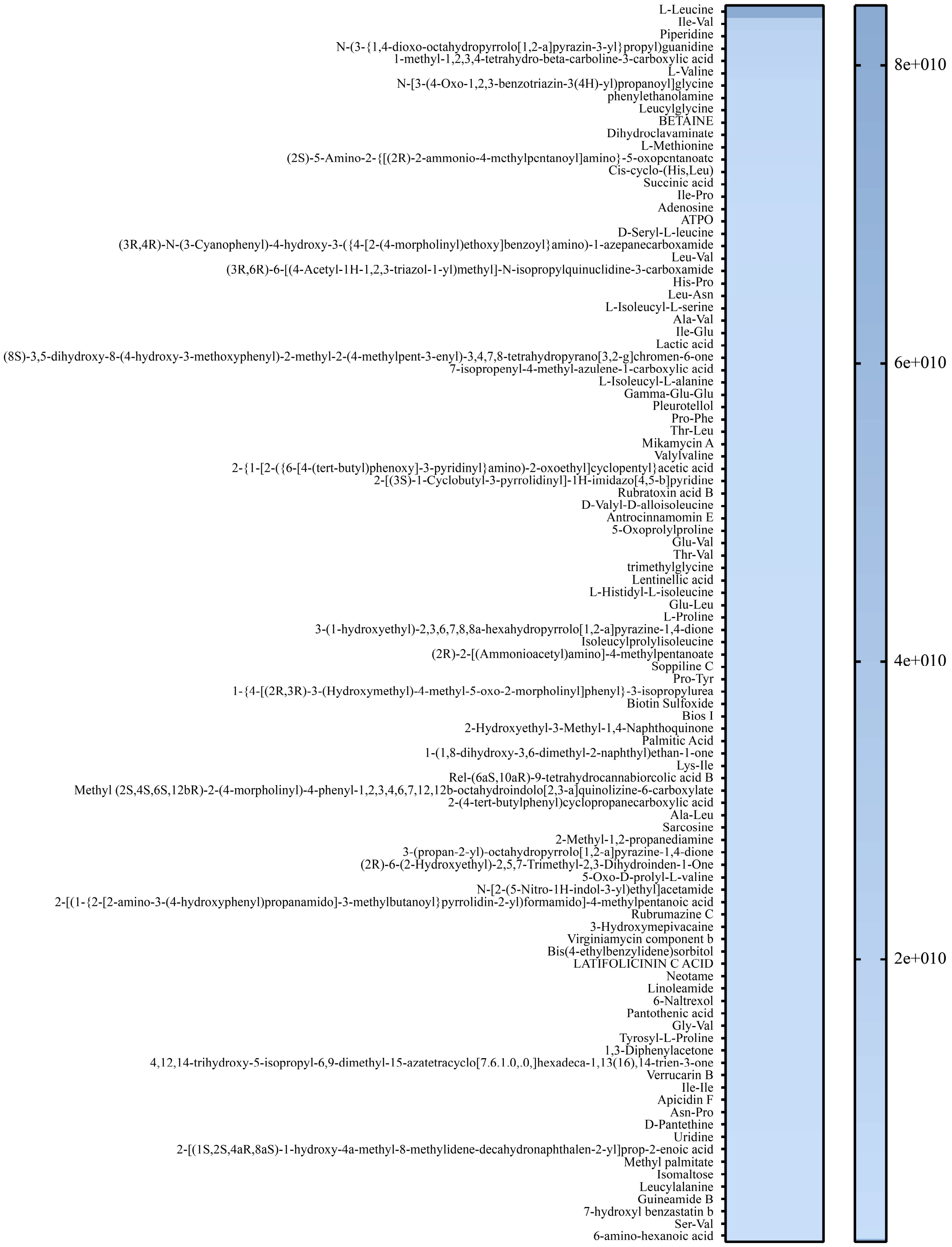

Fig. S8. Metabolome analysis of 1/10 LB medium. The top 100 metabolites of 1/10 LB medium with relatively high abundance were shown on the left. The relative abundance legend was shown on the right.

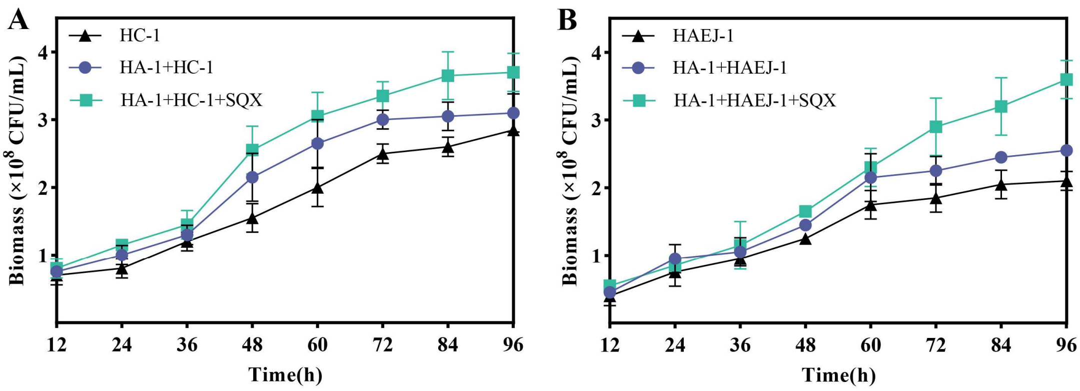

Fig. S9. Growth of strains HC-1 and HAEJ-1 under the conditions of single culture and co-culture. Biomass of (A) strain HC-1 or (B) strain HAEJ-1 cultured alone and co-cultured with strain HA-1 in 1/10 LB medium with or without SQX. Error bars represent the standard deviations of three replicates.

Table S1. The genome-related information of HA-1, HC-1 and HAEJ-1.

Sample Name	Genome Size (bp)	Chromosome	Plasmid	GC Content(%)	CDS	tRNA	rRNA
HA-1	3476900	1	0	69.84	3186	45	6
HC-1	5313991	1	0	35.14	5303	97	7
HAEJ-1	4903006	1	0	67.62	4417	49	3

Table S2. The antibiotic resistance gene information of HA-1, HC-1 and HAEJ-1.

Sample Name	Gene ID	ARO Name	Drug Class	AMR Gene Family	Identity(%)	Coverage(%)
HA-1	gene0309	sul2	sulfonamide antibiotic	sulfonamide resistant sul	100	100
	gene0364	sul1	sulfonamide antibioti	sulfonamide resistant sul	95.8	97.3
	gene0694	folP	sulfonamide antibiotic	dapsone resistant dihydropteroate synthase folP	54.9	64.3
	gene1120	sul1	sulfonamide antibiotic	sulfonamide resistant sul	99.5	100
	gene1122	sul1	sulfonamide antibiotic	sulfonamide resistant sul	95.8	80.9
HC-1	gene0754	MexR	Multiclass antibiotics	resistance-nodulation-cell division (RND) antibiotic efflux pump	24.3	74.1
	gene5197	sul4	sulfonamide antibiotic	sulfonamide resistant sul	50.4	89.2
HAEJ-1	gene0138	CpxR	Multiclass antibiotics	resistance-nodulation-cell division (RND) antibiotic efflux pump	32.5	70.7
	gene0693	folp	sulfonamide antibiotic	dapsone resistant dihydropteroate synthase folP	62.4	88.3
	gene0977	nalD	Multiclass antibiotics	resistance-nodulation-cell division (RND) antibiotic efflux pump	31	38
	gene1283	nalC	Multiclass antibiotics	resistance-nodulation-cell division (RND) antibiotic efflux pump	43.5	26.6
	gene1734	nalD	Multiclass antibiotics	resistance-nodulation-cell division (RND) antibiotic efflux pump	39	28
	gene2843	sul4	sulfonamide antibiotic	sulfonamide resistant sul	37.3	90.4
